# Genome-Wide Insights Into the Organelle Translocation of Photosynthetic NDH-1 Genes During Evolution

**DOI:** 10.3389/fmicb.2022.956578

**Published:** 2022-07-13

**Authors:** Jie Yu, Zhaoxing Ran, Jingsong Zhang, Lanzhen Wei, Weimin Ma

**Affiliations:** College of Life Sciences, Shanghai Normal University, Shanghai, China

**Keywords:** organelle translocation, photosynthetic NDH-1, mitochondrial NDH-1, evolutionary events, plant evolution

## Abstract

Translocation of chloroplast-located genes to mitochondria or nucleus is considered to be a safety strategy that impedes mutation of photosynthetic genes and maintains their household function during evolution. The organelle translocation strategy is also developed in photosynthetic NDH-1 (pNDH-1) genes but its understanding is still far from complete. Here, we found that the mutation rate of the conserved pNDH-1 genes was gradually reduced but their selection pressure was maintained at a high level during evolution from cyanobacteria to angiosperm. By contrast, oxygenic photosynthesis-specific (OPS) pNDH-1 genes had an opposite trend, explaining the reason why they were transferred from the reactive oxygen species (ROS)-enriched chloroplast to the ROS-barren nucleus. Further, genome-wide sequence analysis supported the possibility that all conserved pNDH-1 genes lost in chloroplast genomes of Chlorophyceae and Pinaceae were transferred to the ROS-less mitochondrial genome as deduced from their truncated pNDH-1 gene fragments. Collectively, we propose that the organelle translocation strategy of pNDH-1 genes during evolution is necessary to maintain the function of the pNDH-1 complex as an important antioxidant mechanism for efficient photosynthesis.

## Introduction

During evolution of photosynthetic organisms, the rise of O_2_ and environmental stress inevitably results in the production of reactive oxygen species (ROS), which can trigger the mutation of genes under the low selection pressure (Ishikawa et al., 2008; Otten and Smeets, 2015). It is well known that in eukaryotic photosynthetic organisms, genes are located in the genomes of chloroplast, mitochondria, and nucleus. Considering the O_2_ content, the ROS concentrations of chloroplast, mitochondria, and nucleus under environmental stresses are assumed to be relatively abundant, less, and barren, respectively (Allen and Raven, 1996; Martin and Herrmann, 1998; Adams and Palmer, 2003; Foyer and Noctor, 2003; Laloi et al., 2004; Zhao et al., 2020). As a consequence, the organelle translocation is considered to be an important safety strategy that impedes mutation of photosynthetic genes and maintains their household function during evolution.

Complete sequencing of the chloroplast genomes of *Marchantia polymorpha* and *Nicotiana tabacum* unexpectedly demonstrates the presence of photosynthetic NDH-1 (pNDH-1) (hereafter referred to as pNDH-1) genes (Ohyama et al., 1986; Shinozaki et al., 1986). All complexes of pNDH-1 are involved in cyclic electron transfer around photosystem I (Bernát et al., 2011), which is an important antioxidant mechanism that balances the ATP/NADPH ratio required for the Calvin–Benson cycle and reduces the ROS production (Arnon, 1971; Kramer and Evans, 2011). They consist of conserved subunits (NdhA to NdhK) and oxygenic photosynthesis-specific (OPS) subunits (such as NdhL to NdhQ, NdhS, and NdhV) (Laughlin et al., 2019; Schuller et al., 2019, 2020; Pan et al., 2020; Zhang et al., 2020; Shen et al., 2022). It is found that these conserved genes of pNDH-1 reside in chloroplast genome (Ohyama et al., 1986; Shinozaki et al., 1986) but its OPS genes are translocated to the nucleus genome (Rumeau et al., 2005; Ishikawa et al., 2008; Shimizu et al., 2008; Suorsa et al., 2009; Yamamoto et al., 2011; Fan et al., 2015). However, little is known regarding the reason why these OPS pNDH-1 genes are translocated to the nucleus genome. In addition, genome-wide sequence analysis of pNDH-1 genes in Chlorophyceae and Pinaceae indicates that OPS pNDH-1 genes reside also in the nucleus genome, but all conserved pNDH-1 genes are lost entirely in the chloroplast and nucleus genomes (Wakasugi et al., 1994; Maul et al., 2002; Nystedt et al., 2013; Neale et al., 2014; Ranade et al., 2016; Lin et al., 2017). However, as yet, whether all these conserved pNDH-1 genes have been lost entirely or transferred to the mitochondrial genome remains a mystery.

Here, we calculated and analyzed the mutation frequency and selection pressure, explaining the reason why these OPS pNDH-1 genes were transferred from chloroplast to the nucleus. Further, we found the presence of conserved pNDH-1 gene fragments in mitochondrial genomes of Chlorophyceae and Pinaceae, implying that these conserved pNDH-1 genes lost in chloroplast genomes of Chlorophyceae and Pinaceae were transferred to their mitochondrial genomes. Collectively, our data provide new insights into the organelle translocation of pNDH-1 genes during evolution from cyanobacteria to angiosperm.

## Materials and Methods

### Phylogenetic Analysis

Phylogenetic tree was constructed based on *rbcL* gene of Methanogen, Cyanobacteria, Chlorophyceae, Bryophyta, Pinaceae, Monocots, and Dicots. The gene sequences of *rbcL* from different species were obtained from the National Center for Biotechnology Information (NCBI^1^). The names of the selected species and their GenBank accession numbers are listed in Supplementary Table 1. Sequence alignments were performed using MUSCLE (Edgar, 2004). The aligned dataset was analyzed in Data Analysis in Molecular Biology and Evolution (DAMBE) version 7 (Xia, 2018), and was converted into MEGA format. Unrooted phylogenetic trees were created using MEGA version 7 (Kumar et al., 2016) and maximum likelihood method (Felsenstein, 1981) with the bootstrap support of 1,000 replicates. Creating the phylogenetic tree, the parameters used were: complete deletion of gaps/missing data, distance model set to applying the nucleotide kimura-2-parameter, homogeneous pattern among lineages and uniform rates among sites and using the maximum composite likelihood model. The FigTree (v1.3.1^2^) was used for the unrooted phylogenetic tree visualization.

### Calculation of Synonymous Substitution Rate and Non-synonymous Substitution Rate

The values of synonymous substitution rate (*d*_S_) and non-synonymous substitution rate (*d*_N_) were calculated using DNAsp6 software (Rozas et al., 2017). We first removed the terminators of each sequence and then used MUSCLE for sequence alignment, and the alignments of all these genes of pNDH-1 and rNDH-1 were converted into a codon alignment using TranslatorX (Abascal et al., 2010). The ambiguously aligned regions were excluded using trimAl v1.2 (Capella-Gutiérrez et al., 2009) and the results were exported as a Fasta file. Having opened the exported file with DNAsp6, we set the genomic state and chromosomal location, assigned the coding regions, and calculated the *d*_S_ and *d*_N_ values. The average values were calculated using SigmaPlot 14.0.

### Calculation of Amino Acid Conservative Substitution

Calculation of amino acid conservative substitution (*K*_c_) was carried out with the aid of a pipeline SAMEM v.0.83.^3^ The SAMEM package (Gunbin et al., 2011) has a major path for the gene evolution analysis. We divided these 20 amino acids into two groups according to their physicochemical properties, such as volume (RQEHILKMFWYV ANDCGPST), which are related to protein function. Amino acid substitutions within groups are called conservative substitutions (Hanada et al., 2007). The general step and method of calculating *K*_c_ are as follows: translation of nucleic acid sequence into amino acid sequence by Transeq, multiple alignment of amino acid sequences by the Mafft 6.717 algorithm (Katoh and Toh, 2008) using the BLOSUM 62 matrix (Henikoff and Henikoff, 1992), and models of amino acid substitutions were calculated based on multiple alignment using the Modelestimator algorithm (Arvestad, 2006), phylogenetic trees were calculated on the basis of the replacement model using FastTree 2.1.1 (Price et al., 2010), ancestral gene sequences are reconstructed based on gapless alignments of codons using FASTML (Pupko et al., 2002), calculation of *K*_c_ using Zhang’s (2000) method (the HON-NEW program). For each of 531 properties (Kawashima et al., 2008), amino acids are divided into classes by k-means clustering using R.

### Sequence Analysis

Homology search was performed by comparing amino acid sequences with sequences in local nucleotide databases (TBLASTN). Eleven *Arabidopsis thaliana* pNDH-1 gene sequences were used as templates for TBLASTN searches of Chlorophyceae and Pinaceae mitochondrial genomes. The TBLASTN expectation value threshold E was altered to 10 to allow for a less stringent alignment search.

### Data Availability

The data underlying this article are available within the NCBI GenBank database,^4^ and all GenBank accession numbers are listed in Supplementary Table 1. The alignments analyzed in this study are available in the article’s online Supplementary Figures 2–4.

## Results

### Phylogenetic Tree Marks These Important Evolutionary Events of Photosynthetic NDH-1

**FIGURE 1:**
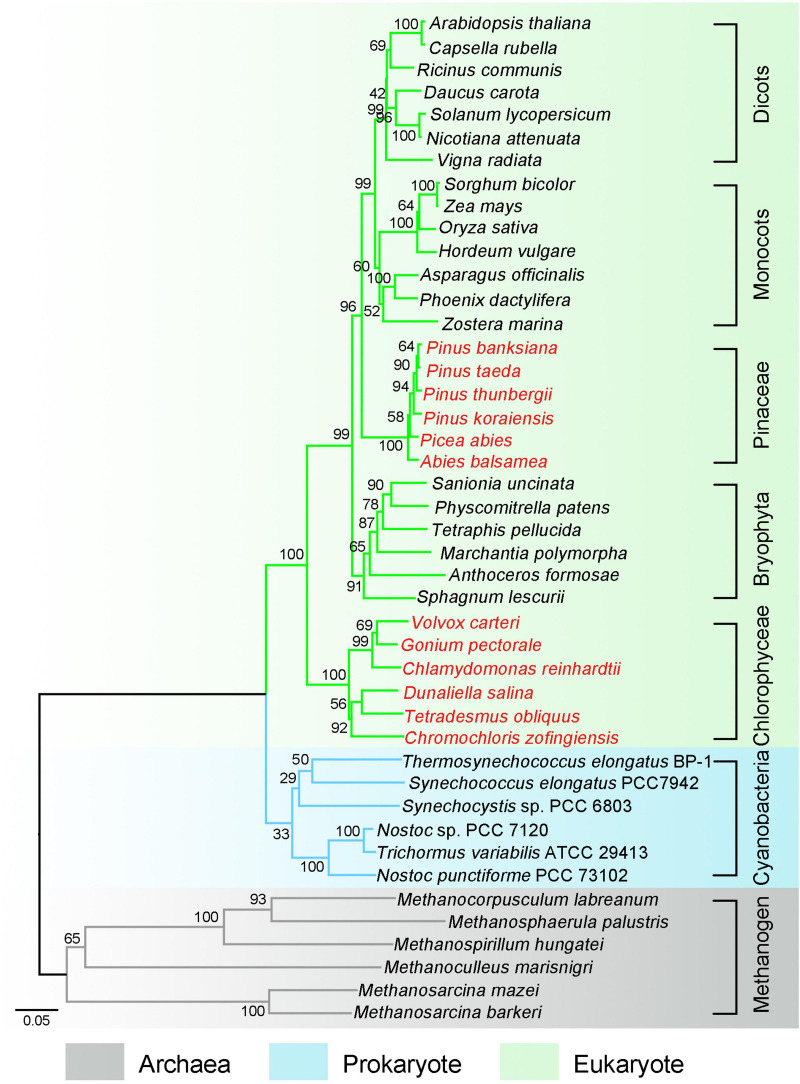
Phylogenetic tree marks these important events of photosynthetic NDH-1 (pNDH-1) during evolution from archaea to eukaryote. Phylogenetic tree was constructed based on marker gene *rbcL*. The pNDH-1 originates from a group 4 membrane-bound [NiFe] hydrogenase and evolves from archaea (gray tree branches) to prokaryote (blue tree branches) and to eukaryotic photosynthetic organisms (green tree branches). In chloroplast, the species with pNDH-1 are represented by black letters, and the species without pNDH-1 are represented by red letters.

Genome-wide sequence analysis suggests that pNDH-1 originates from a group 4 membrane-bound [NiFe] hydrogenase (Böhm et al., 1990; Peltier et al., 2016) and evolves from archaea (gray tree branches in Figure 1) to prokaryote (blue tree branches in Figure 1) and to eukaryotic photosynthetic organisms (green tree branches in Figure 1). In eukaryotic photosynthetic organisms except Chlorophyceae, Pinaceae and Orchidaceae, conserved pNDH-1 genes reside in chloroplast genome, whereas OPS pNDH-1 genes are transferred to the nucleus genome (Rumeau et al., 2005; Ishikawa et al., 2008; Shimizu et al., 2008; Suorsa et al., 2009; Yamamoto et al., 2011; Fan et al., 2015). In addition, in eukaryotic Chlorophyceae and Pinaceae (red species name in Figure 1), OPS pNDH-1 genes reside also in the nucleus genome, but all conserved pNDH-1 genes are lost entirely in their chloroplast and nucleus genomes (Wakasugi et al., 1994; Maul et al., 2002; Lin et al., 2017). The below investigations will try to explain the reason why these OPS pNDH-1 genes in eukaryotic photosynthetic organisms except Chlorophyceae, Pinaceae, and Orchidaceae were transferred from chloroplast genome to the nucleus genome and unravel the mystery whether conserved pNDH-1 genes of Chlorophyceae and Pinaceae have been lost entirely or transferred to the mitochondrial genome.

### An Evolutionary Trend of Conserved and Oxygenic Photosynthesis-Specific Photosynthetic NDH-1 Genes

**FIGURE 2:**
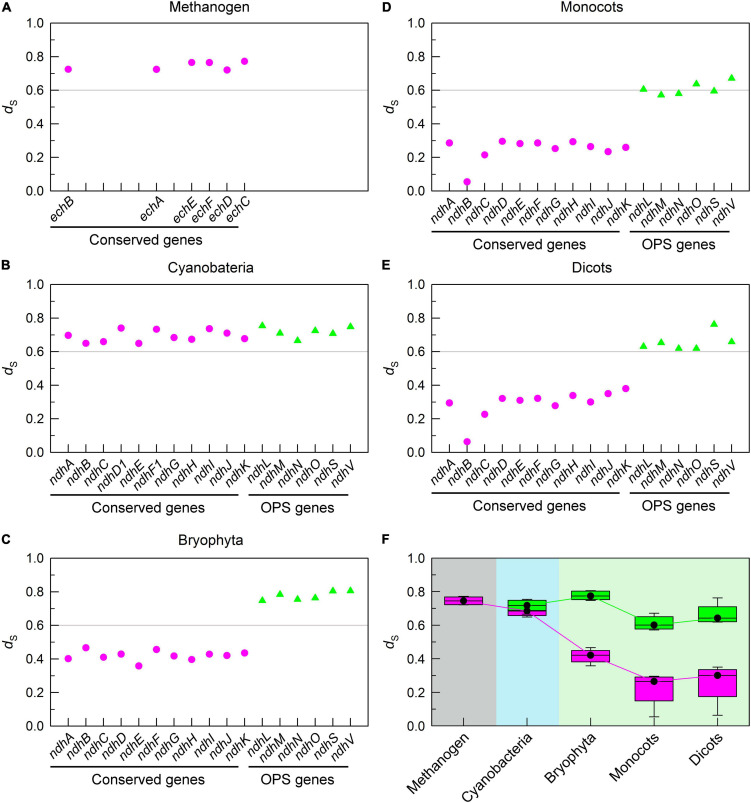
The trend of mutation rate of pNDH-1 genes during evolution from cyanobacteria to dicots. The mutation rate is deduced from the values of synonymous substitution rate (*d*_S_). The Scatter plot shows the respective *d*_S_ values of conserved genes and oxygenic photosynthesis-specific (OPS) genes of pNDH-1 in methanogen **(A)**, cyanobacteria **(B)**, bryophyta **(C)**, monocots **(D)**, and dicots **(E)**. The pink dots represent conserved genes and the green triangles represent OPS genes. Box plot shows the average *d*_S_ values of conserved genes and OPS genes of pNDH-1 in methanogen, cyanobacteria, bryophyta, monocots, and dicots **(F)**. The box represents the values between the quartiles and the black lines inside the box represent the median value.

During the evolution process from cyanobacteria to dicots, we found that the mutation rate of conserved pNDH-1 genes was gradually decreased, as deduced from the results of *d*_S_ (Figures 2A–F). This was supported by the data of *K*_c_ (Supplementary Figure 1). As a consequence, the mutation rate of conserved pNDH-1 genes has a trend of gradual decrease during evolution.

**FIGURE 3:**
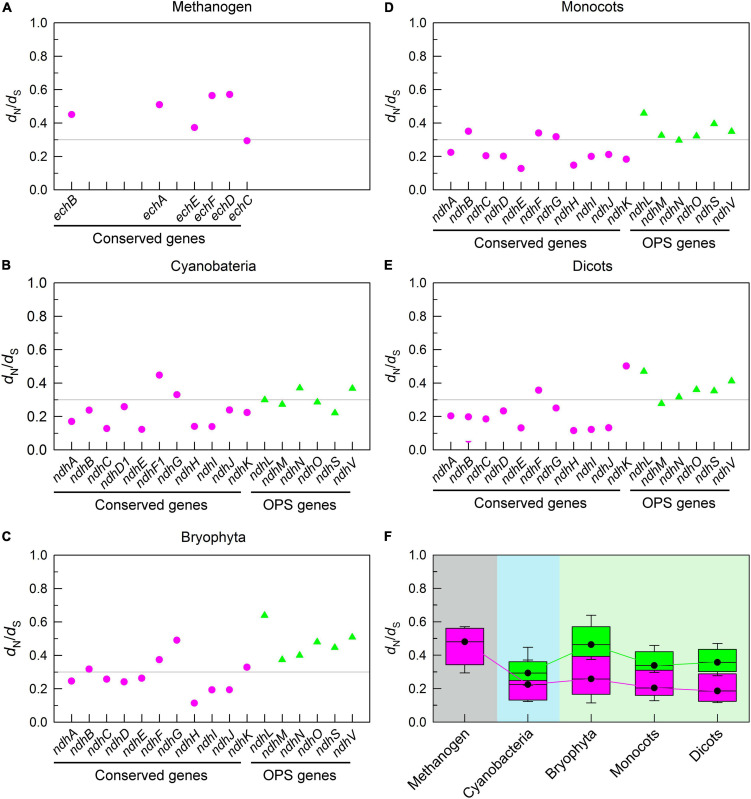
The trend of selection pressure of pNDH-1 genes during evolution from cyanobacteria to dicots. The selection pressure is deduced from the values of non-synonymous substitution rate (*d*_N_)/*d*_S_. Scatter plot shows the respective *d*_N_/*d*_S_ values of conserved genes and OPS genes of pNDH-1 in methanogen **(A)**, cyanobacteria **(B)**, bryophyta **(C)**, monocots **(D)**, and dicots **(E)**. The pink dots represent conserved genes and the green triangles represent OPS genes. Box plot shows the average *d*_N_/*d*_S_ values of conserved genes and OPS genes of pNDH-1 in methanogen, cyanobacteria, bryophyta, monocots, and dicots **(F)**. The box represents the values between the quartiles and the black lines inside the box represent the median value.

To fully understand the trend of conserved pNDH-1 genes during evolution, we calculated the ratio of *d*_N_ to *d*_S_. Our data indicated that all *d*_N_/*d*_S_ ratios of conserved pNDH-1 genes were less than 1 (Figures 3A–F), indicating that the evolutional rate of all these conserved pNDH-1 genes was relatively slow and under the purifying selection (Endo et al., 1997; Messier and Stewart, 1997). Further, under the purifying selection, the magnitude of *d*_N_/*d*_S_ values could reflect the selection pressure of these conserved pNDH-1 genes (Berg and Kurland, 2000). If the *d*_N_/*d*_S_ ratio was more close to 0, the selection pressure was higher, whereas if the ratio was more close to 1, the selection pressure was lower. We found that the selection pressure of conserved pNDH-1 genes was increased during evolution from cyanobacteria to dicots, as deduced from the results of *d*_N_/*d*_S_ values (Figures 3A–F). Collectively, we propose that conserved pNDH-1 genes have an evolutionary trend that the mutation rate was gradually decreased but the selection pressure was maintained at a relatively high level.

Unexpectedly, the mutation rate of OPS pNDH-1 genes was increased and kept at a high level during evolution from cyanobacteria to dicots, as deduced from the *d*_S_ values (Figures 2A–F). Meanwhile, the selection pressure of OPS pNDH-1 genes was deceased during evolution from cyanobacteria to dicots, as deduced from the data of *d*_N_/*d*_S_ values (Figures 3A–F). Taking all these results together, we can clearly find that OPS and conserved pNDH-1 genes have a distinctly different trend of their mutation rate and selection pressure during evolution.

### Presence of Photosynthetic NDH-1 Gene Fragments in the Mitochondrial Genomes of Chlorophyceae and Pinaceae

**FIGURE 4:**
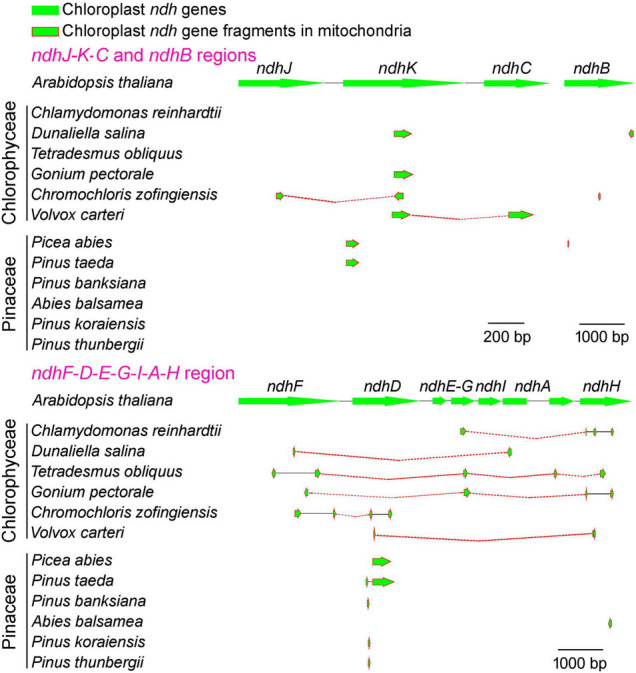
Comparison of Chlorophyceae and Pinaceae mitochondrial genomes with *Arabidopsis thaliana* chloroplast pNDH-1 genes. The arrows indicate coding regions. The arrowheads indicate the direction of the genes.

To unravel the mystery whether these conserved pNDH-1 genes have been lost entirely or transferred to the mitochondrial genome, we conducted the sequence searches in the mitochondrial genomes of Chlorophyceae and Pinaceae using pNDH-1 sequences of *Arabidopsis thaliana* as templates. Our results revealed that the fragments of conserved pNDH-1 genes lost in the chloroplast genomes of Chlorophyceae and Pinaceae were found to be in their mitochondrial genomes (Figure 4 and Supplementary Figures 2–4). Collectively, we propose that these conserved pNDH-1 genes lost in the chloroplast genomes of Chlorophyceae and Pinaceae were transferred to their mitochondrial genomes.

### A Trend of Respiratory NDH-1 Genes During Evolution

**FIGURE 5:**
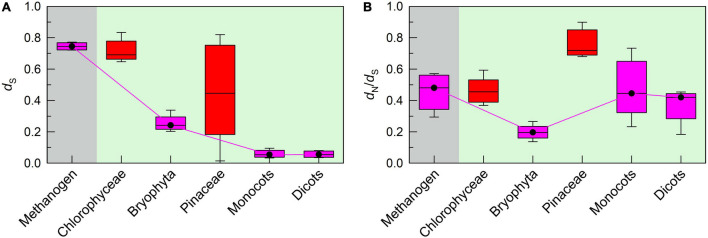
The trends of mutation rate and selection pressure of rNDH-1 genes during evolution from chlorophyceae to dicots. The mutation rate and selection pressure are deduced from the values of *d*_S_ and *d*_N_/*d*_S_, respectively. Boxplots show the average *d*_S_ **(A)** and *d*_N_/*d*_S_ **(B)** values of rNDH-1 in methanogen, Chlorophyceae, bryophyta, Pinaceae, monocots, and dicots. The box represents the values between the quartiles and the black lines inside the box represent the median value.

Consistent with the conserved pNDH-1 genes, the mutation rate of respiratory NDH-1 (rNDH-1) genes was gradually decreased but their selection pressure was maintained at a relatively high level during evolution from methanogen to dicots (pink in Figures 5A,B). However, the mutation rate and selection pressure of rNDH-1 genes in Chlorophyceae and Pinaceae did not follow the trend of rNDH-1 during evolution from methanogen to dicots (red in Figures 5A,B). It is reasonable to infer that the transfer of chloroplast pNDH-1 genes of Chlorophyceae and Pinaceae to their mitochondrial genomes results in mitochondrial DNA rearrangement, thereby increasing the mutation rate of rNDH-1 genes and relatively decreasing their selection pressure. This supports the conclusion that these conserved pNDH-1 genes lost in chloroplast genomes of Chlorophyceae and Pinaceae were transferred to their mitochondrial genomes.

## Discussion

During the evolution from prokaryotic cyanobacteria to eukaryotic angiosperm, organelle translocation is considered to be an important safety strategy that impedes mutation of photosynthetic genes and maintain their household function (Baldauf and Palmer, 1990; Gantt et al., 1991; Martin et al., 1998; Adams and Palmer, 2003; Rokka et al., 2005). Such organelle translocation is also found to be a universal safety strategy, for example, the mitochondrial genome contains between 1 and 10% of chloroplast sequences in different seed plants (Stern and Lonsdale, 1982; Joyce and Gray, 1989; Wang et al., 2007, 2012). In eukaryotic photosynthetic organisms except Chlorophyceae, Pinaceae, and Orchidaceae, OPS pNDH-1 genes are found to transfer from chloroplast genome to the nucleus genome during evolution (Rumeau et al., 2005; Ishikawa et al., 2008; Shimizu et al., 2008; Suorsa et al., 2009; Yamamoto et al., 2011; Fan et al., 2015), although conserved pNDH-1 genes still reside in chloroplast genome (Ohyama et al., 1986; Shinozaki et al., 1986).

The results of this study found that two distinctly different strategies have been developed by conserved and OPS pNDH-1 genes to impede their mutations and maintain their functions (Figures 2, 3 and Supplementary Figure 1). Conserved pNDH-1 genes develop a safety strategy *via* decreasing their mutation rate and increasing their selection pressure, while OPS pNDH-1 genes develop another safety strategy *via* transferring them from the chloroplast genome to a relatively safe nucleus genome (Figures 2, 3 and Supplementary Figure 1). It is worthy of note that according to Mahler’s ratchet effect (Muller, 1964), gene recombination is lacked in chloroplast because of no sexual reproduction. When a gene is successfully transferred from chloroplast to nuclear, in other words, from asexual to sexual, gene recombination is restored and provides a chance to get rid of the fate crisis of gene mutation, reinforcing the conclusion that the nucleus is much safer than the chloroplast. Collectively, during evolution of photosynthetic organisms, these two distinctly different strategies jointly maintain the function of pNDH-1 as an important antioxidant mechanism for efficient photosynthesis through impeding mutation of its conserved and OPS genes.

Consistent with the previously reported Orchidaceae (Lin et al., 2015, 2017), these conserved pNDH-1 genes lost in Chlorophyceae and Pinaceae were transferred from chloroplast genome to the mitochondrial genome as deduced from their common gene fragments (Figure 4 and Supplementary Figures 2–4) and the abnormal mutation rate and selection pressure of rNDH-1 genes (Figure 5). It has been reported that Chlorophyceae green algae frequently meet with various environmental challenges, such as fluctuations in nutrient, light availability, and temperature, in their natural habitat (Varshney et al., 2015). Consistent with this situation, the chloroplast DNA and mitochondrial DNA of Chlorophyceae underwent substantial changes in their architecture (such as gene losses and genome expansion in the case of mitochondrial DNA) during evolution (Turmel et al., 2002; Wodniok et al., 2011). Under this background, it is reasonable to infer that these conserved pNDH-1 genes of Chlorophyceae are lost in chloroplast and are transferred to the mitochondrial genome.

Compared with the land plants, Bryophyta and Pteridophyte, the trees in Pinaceae grow quite high (Graham et al., 1995), implying that they have an efficient photosynthesis and produce more O_2_. Consistent with the hypothesis, Pinaceae underwent an O_2_-rise phase (Savard et al., 1994; Berner, 2006) and may produce abundant ROS in chloroplast but less ROS in mitochondria under environmental stresses (Foyer and Noctor, 2003; Laloi et al., 2004; Zhao et al., 2020). As a consequence, it appears plausible that pNDH-1 genes lost in Pinaceae are transferred from O_2_-enriched chloroplast to the O_2_-less mitochondria as oxygen-consuming organelle.

Based on the aforementioned analysis, we propose that translocation of pNDH-1 genes from chloroplast genome to the nucleus genome or mitochondrial genome is important to maintain the architecture and household function of pNDH-1 during evolution. As a consequence, the function of pNDH-1 as an important antioxidant mechanism can reduce ROS production necessary for the survival of eukaryotic photosynthetic organisms in aerobic environment.

## Data Availability Statement

The raw data supporting the conclusions of this article will be made available by the authors, without undue reservation.

## Author Contributions

WM conceived and designed the project. JY and JZ performed the bioinformatics analysis. JY, ZR, LW, and WM interpreted the data and wrote the manuscript. All authors read and approved the final manuscript.

## Conflict of Interest

The authors declare that the research was conducted in the absence of any commercial or financial relationships that could be construed as a potential conflict of interest.

## Publisher’s Note

All claims expressed in this article are solely those of the authors and do not necessarily represent those of their affiliated organizations, or those of the publisher, the editors and the reviewers. Any product that may be evaluated in this article, or claim that may be made by its manufacturer, is not guaranteed or endorsed by the publisher.

## Footnotes

1
http://www.ncbi.nlm.nih.gov/

2
http://tree.bio.ed.ac.uk/software/figtree

3
http://pixie.bionet.nsc.ru/samem/

4
https://www.ncbi.nlm.nih.gov/genbank

## Abbreviations

*d*_N_: non-synonymous substitution rate
*d*_S_: synonymous substitution rate
*K*_c_: amino acid conservative substitution
OPS: oxygenic photosynthesis-specific
pNDH-1: photosynthetic NDH-1
rNDH-1: respiratory NDH-1
ROS: reactive oxygen species.
