# Supplementary material for: Genome-Wide Insights Into the Organelle Translocation of Photosynthetic NDH-1 Genes During Evolution
Source: Front Microbiol. 2022 Jul 13;13:956578. doi: 10.3389/fmicb.2022.956578 (PMC9326235; doi:10.3389/fmicb.2022.956578)
Supplement: Supplementary file 1 [file Data_Sheet_1.pdf]

Genome-Wide Insights Into the Organelle Translocation of Photosynthetic NDH-1 Genes During Evolution

Jie Yu, Zhaoxing Ran, Jingsong Zhang, Lanzhen Wei*, Weimin Ma*

College of Life Sciences, Shanghai Normal University, Shanghai, China

***Correspondence:** Weimin Ma (wma@shnu.edu.cn) and Lanzhen Wei (weilz@shnu.edu.cn)

This PDF file includes: **Supplementary Figures 1** to **4** and **Supplementary Table 1**.

SUPPLEMENTARY MATERIAL

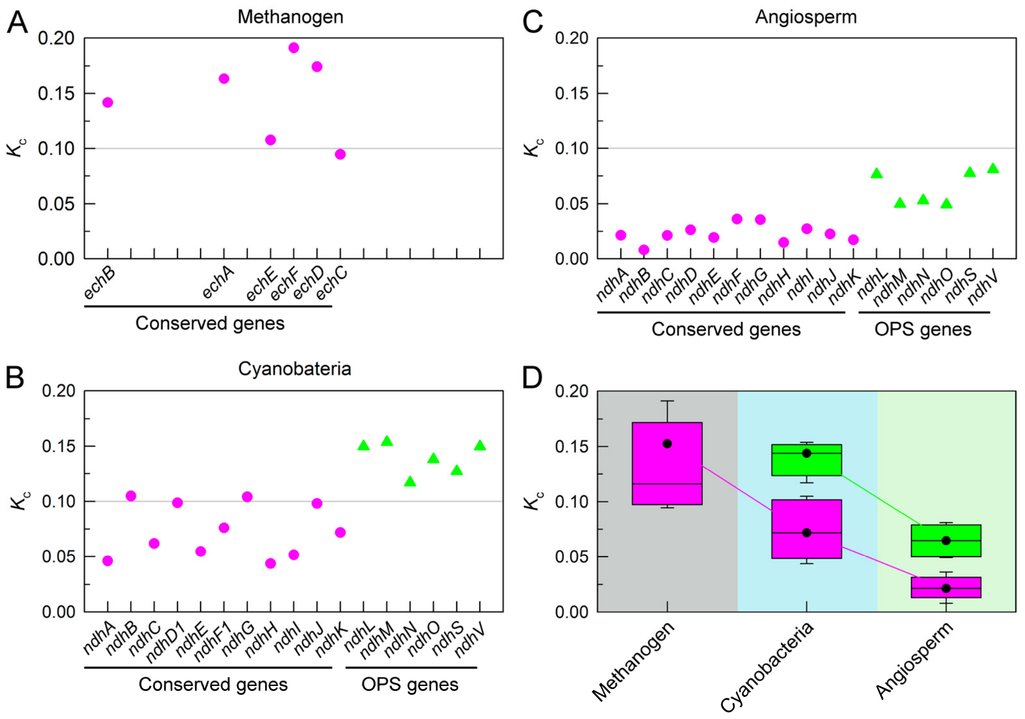

SUPPLEMENTARY FIGURE 1 | The trend of mutation rate of pNDH-1 genes during evolution from cyanobacteria to angiosperm. The mutation rate is deduced from the values of amino acid conservative substitution (K_c). Scatter plot shows the respective K_c values of conserved genes and OPS genes of pNDH-1 in methanogen **(A)** cyanobacteria **(B)**, and angiosperm **(C)**. The pink dots represent conserved genes and the green triangles represent OPS genes. Box plot shows the average K_c values of conserved genes and OPS genes of pNDH-1 in methanogen, cyanobacteria, and angiosperm **(D)**. The box represents the values between the quartiles and the black lines inside the box represent the median value.

ndhJ-K-C region

Chlorophyceae

Dunaliella salina
>*ndhK*
Length = 678

```
Score = 18.5 bits (36), Expect = 4.7, Method: Compositional matrix adjust.
Identities = 12/40 (30%), Positives = 19/40 (48%), Gaps = 7/40 (18%)
Frame = +1

Query  366  YYNISHFHYVLSMGAVFGIFAGLYFWLSLMTGLSYNEARG  405
            Y  +    YV++MGA   I  G++      +  SY+  RG
Sbjct  277  YEQMPEPKYVIAMGAC-TITGGMF------STDSYSTVRG  375
```

Gonium pectorale
>*ndhK*
Length = 678

```
Score = 19.2 bits (38), Expect = 2.6, Method: Compositional matrix adjust.
Identities = 11/37 (30%), Positives = 18/37 (49%), Gaps = 1/37 (3%)
Frame = +1

Query  365  YYVVAHFHYVLSMGAVFGIFGGLYFWGNLITGLGYHE  401
            Y  +    YV++MGA   I GG++   +  T  G  +
Sbjct  277  YEQMPEPKYVIAMGAC-TITGGMFSTDSYSTVRGVDK  384
```

Chromochloris zofingiensis
>*ndhJ*
Length = 477

```
Score = 18.5 bits (36), Expect = 2.9, Method: Compositional matrix adjust.
Identities = 8/14 (57%), Positives = 10/14 (71%), Gaps = 0/14 (0%)
Frame = -3

Query  16   IKVLENGDHNKPKK  29
            I VLEN   +KP+K
Sbjct  340  IPVLENPYFSKPRK  299
```

>*ndhK*
Length = 678

```
Score = 17.7 bits (34), Expect = 3.6, Method: Compositional matrix adjust.
Identities = 7/18 (39%), Positives = 10/18 (56%), Gaps = 0/18 (0%)
Frame = -1

Query  18   PSCQRFGPHNYDVLCFLH  35
            P C   G H+ ++L F H
Sbjct  339  PPCNCTGSHSNNILRFRH  286
```

Volvox carteri
>*ndhK*
Length = 678

```
Score = 20.4 bits (41),  Expect = 1.4, Method: Compositional matrix adjust.
Identities = 11/37 (30%), Positives = 19/37 (51%), Gaps = 1/37 (3%)
Frame = +1

Query  365  YYVVAHFHYVLSMGAVFGIFGGLYFWGNLITGLGYNE  401
            Y  +    YV++MGA   I GG++   +  T  G ++
Sbjct  277  YEQMPEPKYVIAMGAC-TITGGMFSTDSYSTVRGVDK  384
```

```
>ndhC
Length = 363

Score = 19.2 bits (38),  Expect = 1.8, Method: Compositional matrix adjust.
Identities = 12/43 (28%), Positives = 19/43 (44%), Gaps = 5/43 (12%)
Frame = +1

Query  103  YGSGTQPRELVWITGVI-----ILLVMIITAFIGYVLPWGMSF  140
            Y SG +P    W+   I       L+ ++       ++ PW MSF
Sbjct  130  YESGIEPIGDAWLQFRIRYYMFALVFVVFDVETVFLYPWAMSF  258
```

Pinaceae

Picea abies

```
>ndhK
Length = 678

Score = 18.1 bits (35), Expect = 5.7, Method: Compositional matrix adjust.
Identities = 8/24 (33%), Positives = 13/24 (54%), Gaps = 0/24 (0%)
Frame = +1

Query  12  KQPILSTLNQHLIDHPTPSNLSYW  35
           K PIL    ++ +   T ++LS W
Sbjct  13  KFPILDRTTKNSVISTTLNDLSNW  84
```

Pinus taeda

```
>ndhK
Length = 678

Score = 17.7 bits (34), Expect = 6.2, Method: Compositional matrix adjust.
Identities = 8/24 (33%), Positives = 13/24 (54%), Gaps = 0/24 (0%)
Frame = +1

Query  12  KQPILSTLNQHLIDHPTPSNLSYW  35
           K PIL    ++ +   T ++LS W
Sbjct  13  KFPILDRTTKNSVISTTLNDLSNW  84
```

SUPPLEMENTARY FIGURE 2 | Sequence alignment between the mitochondrial genome of Chlorophyceae and Pinaceae and the *ndhJ-K-C* region of *Arabidopsis thaliana*.

ndhB region

Chlorophyceae

Dunaliella salina

```
>ndhB
Length = 1539

Score = 18.9 bits (37), Expect = 2.9, Method: Compositional matrix adjust.
Identities = 11/34 (32%), Positives = 14/34 (41%), Gaps = 0/34 (0%)
Frame = -1

Query  6     KTGPLKVLNNHLNVYPTPMNLSYAYNFGSLAGIV  39
             K G L   NN ++ Y       + YN     GIV
Sbjct  1530  KKGILSNRNNRVH*YSWYSRCYHTYNHTQFDGIV  1429
```

Chromochloris zofingiensis

```
>ndhB
Length = 1539

Score = 16.9 bits (32), Expect = 6.5, Method: Compositional matrix adjust.
Identities = 8/17 (47%), Positives = 9/17 (53%), Gaps = 0/17 (0%)
Frame = -1

Query  101  FSTFRFSSFDWIHESFV  117
            F + R  S DW   SFV
Sbjct  819  FRSNRKKSNDWSGRSFV  769
```

Pinaceae

Picea abies

```
>ndhB
Length = 1539

Score = 17.7 bits (34), Expect = 8.0, Method: Compositional matrix adjust.
Identities = 6/13 (46%), Positives = 10/13 (77%), Gaps = 0/13 (0%)
Frame = +2

Query  245  FSSIWIFYAPNVS  257
            FSS+ + ++ NVS
Sbjct  74   FSSMEVLFSQNVS  112
```

SUPPLEMENTARY FIGURE 3 | Sequence alignment between the mitochondrial genome of Chlorophyceae and Pinaceae and the *ndhB* region of *Arabidopsis thaliana*.

ndhF-D-E-G-I-A-H region

Chlorophyceae

Chlamydomonas reinhardtii

>*ndhG*
Length = 531

Score = 17.7 bits (34), Expect = 7.7, Method: Compositional matrix adjust.
Identities = 12/42 (29%), Positives = 21/42 (50%), Gaps = 2/42 (5%)
Frame = +3

```
Query  399   YHEGRAMVHF-WLLFIGV-NLTFFPQHFLGLAGMPRRMFDYA  438
             +H  +++ H+ W  F+G+ N +FF   +     M R   DY
Sbjct  258   FHRFQSVDHWEWDYFVGLYNYSFFINVYYSRYVMVRGYLDYK  383
```

>*ndhH*
Length = 1123

Score = 19.2 bits (38), Expect = 2.2, Method: Compositional matrix adjust.
Identities = 5/8 (63%), Positives = 8/8 (100%), Gaps = 0/8 (0%)
Frame = +1

```
Query  363   CQPLVGYL  370
             C+P++GYL
Sbjct  118   CEPILGYL  141
```

Score = 18.1 bits (35), Expect = 4.6, Method: Compositional matrix adjust.
Identities = 8/20 (40%), Positives = 11/20 (55%), Gaps = 0/20 (0%)
Frame = +3

```
Query  46    TGILLAMHYVGHVDYAFASV  65
              G L   +Y G   Y+F+SV
Sbjct  288   KGQLYQSNYAGIESYSFSSV  347
```

Score = 17.7 bits (34), Expect = 6.6, Method: Compositional matrix adjust.
Identities = 7/17 (41%), Positives = 9/17 (53%), Gaps = 0/17 (0%)
Frame = +2

```
Query  16    HLVAYPTPMNLNYSWNG  32
              L+     MNLN  +NG
Sbjct  704   KLIVMSLTMNLNGKFNG  754
```

Dunaliella salina

>*ndhF*
Length = 2241

Score = 19.6 bits (39), Expect = 2.4, Method: Compositional matrix adjust.
Identities = 7/15 (47%), Positives = 9/15 (60%), Gaps = 0/15 (0%)
Frame = +3

```
Query  98     NNISFWLNPPAFFLL  112
              N +S W  PP  FL+
Sbjct  1203   NTLSLWYPPPCLFLV  1247
```

>*ndhA*
Length = 1083

Score = 18.1 bits (35), Expect = 7.4, Method: Compositional matrix adjust.
Identities = 9/28 (32%), Positives = 15/28 (54%), Gaps = 2/28 (7%)
Frame = -2

```
Query  479   PSASSSLEWLLPA--TPANHTFNQLPVL  504
             P+ S  +   +PA  + +NHT    PV+
Sbjct  203   PAYSGPIRCCIPADISLSNHTITSTPVM  120
```

Tetradesmus obliquus

```
>ndhF
Length = 2241

Score = 19.2 bits (38), Expect = 3.5, Method: Compositional matrix adjust.
Identities = 12/37 (32%), Positives = 19/37 (51%), Gaps = 1/37 (3%)
Frame = +1

Query  348   MLFALGFLVLFTIG-GLTGIVLANAGVDVALHDTYYV  383
             +LF +  LVLFT+  G  GI     G+D  +   ++
Sbjct  1639  ILFPMLILVLFTLFIGAIGIPFNQEGIDFDILSKFFT  1749

Score = 18.9 bits (37), Expect = 4.9, Method: Compositional matrix adjust.
Identities = 9/23 (39%), Positives = 14/23 (61%), Gaps = 1/23 (4%)
Frame = -2

Query  39   ATTMSLLIRMELRSTGPGILAGN  61
            AT ++  IR E+   GP + +GN
Sbjct  812  ATIVAACIRAEM-GVGPSMASGN  747
```

```
>ndhG
Length = 531

Score = 19.2 bits (38), Expect = 3.1, Method: Composition-based stats.
Identities = 11/28 (39%), Positives = 16/28 (57%), Gaps = 1/28 (4%)
Frame = +3

Query  427  FFLGV-NLTFFPQHFLGLAGMPRRYLDY  453
            +F+G+ N +FF   +     M R YLDY
Sbjct  297  YFVGLYNYSFFINVYYSRYVMVRGYLDY  380
```

```
>ndhA
Length = 1083

Score = 17.7 bits (34), Expect = 7.0, Method: Compositional matrix adjust.
Identities = 7/18 (39%), Positives = 11/18 (61%), Gaps = 0/18 (0%)
Frame = +3

Query  186  RFYSFHYLFPFILRRLSL  203
            RFY F   FP  +R +++
Sbjct  639  RFYHFSNFFPSRMREVTV  692
```

```
>ndhH
Length = 1123

Score = 18.5 bits (36), Expect = 3.7, Method: Compositional matrix adjust.
Identities = 10/36 (28%), Positives = 16/36 (44%), Gaps = 0/36 (0%)
Frame = +1

Query  70   FHSVQHLMRDVPNGWLLRYLHANGRSLFFIVVYMHL  105
            F  +  +  D+P GW+ + L      L  +V Y  L
Sbjct  469  FFRIGGIAADLPYGWIDKCLDFCDYFLTEVVEYQKL  576
```

Gonium pectorale

```
>ndhF
Length = 2241

Score = 18.1 bits (35), Expect = 5.9, Method: Compositional matrix adjust.
Identities = 8/24 (33%), Positives = 13/24 (54%), Gaps = 0/24 (0%)
Frame = -3

Query  159   DAIKYWLWGGFSIDQPTLNRFYSL  182
             D + Y+L+  F   +  L  +YSL
Sbjct  1564  DFLHYYLFYMFYCKKKKLFLYYSL  1493
```

>*ndhG*
Length = 531

```
Score = 17.7 bits (34), Expect = 7.7, Method: Compositional matrix adjust.
Identities = 12/42 (29%), Positives = 21/42 (50%), Gaps = 2/42 (5%)
Frame = +3

Query  399  YHEGRAMVHF-WLLFIGV-NLTFFPQHFLGLAGMPRRMFDYA  438
            +H  +++ H+ W  F+G+ N +FF   +     M R   DY
Sbjct  258  FHRFQSVDHWEWDYFVGLYNYSFFINVYYSRYVMVRGYLDYK  38
```

>*ndhH*
Length = 1123

```
Score = 19.2 bits (38), Expect = 2.3, Method: Compositional matrix adjust.
Identities = 5/8 (63%), Positives = 8/8 (100%), Gaps = 0/8 (0%)
Frame = +1

Query  363  CQPLVGYL  370
            C+P++GYL
Sbjct  118  CEPILGYL  141

Score = 17.7 bits (34), Expect = 6.2, Method: Compositional matrix adjust.
Identities = 7/17 (41%), Positives = 9/17 (53%), Gaps = 0/17 (0%)
Frame = +2

Query  16   HLVAYPTPMNLNYSWNG  32
             L+     MNLN  +NG
Sbjct  704  KLIVMSLTMNLNGKFNG  754
```

Chromochloris zofingiensis

>*ndhF*
Length = 2241

```
Score = 17.3 bits (33), Expect = 4.5, Method: Compositional matrix adjust.
Identities = 11/44 (25%), Positives = 18/44 (41%), Gaps = 2/44 (5%)
Frame = +1

Query  155   DTKFFNPTDVEFLCQT--LTHKYGWKVSINKHKNHCVLYIWKVS  196
             D+  F+P      C T  LT  Y +++ +   + H   Y    S
Sbjct  1267  DSLLFSPIFAIIACSTAGLTAFYMFRIYLLTFEGHLNTYFLNYS  1398

Score = 16.9 bits (32), Expect = 6.1, Method: Compositional matrix adjust.
Identities = 6/14 (43%), Positives = 8/14 (57%), Gaps = 0/14 (0%)
Frame = +3

Query  165   EFLCQTLTHKYGWK  178
             +FLC+     Y WK
Sbjct  2112  KFLCRRSNKIYRWK  2153
```

>*ndhD*
Length = 1503

```
Score = 16.9 bits (32), Expect = 5.9, Method: Compositional matrix adjust.
Identities = 7/22 (32%), Positives = 12/22 (55%), Gaps = 0/22 (0%)
Frame = +3

Query  6    MIICSAFYVPKIPSCQRFGPHN  27
            +I   +FYV  +    R+ P+N
Sbjct  801  IITPRSFYVFSLVVGSRYNPNN  866

Score = 17.3 bits (33), Expect = 8.0, Method: Compositional matrix adjust.
Identities = 6/7 (86%), Positives = 7/7 (100%), Gaps = 0/7 (0%)
Frame = +3

Query  245   NLYSYTH  251
             NLYS+TH
Sbjct  1395  NLYSFTH  1415
```

Volvox carteri

```
>ndhD
Length = 1503

Score = 15.4 bits (28),  Expect = 8.7, Method: Compositional matrix adjust.
Identities = 7/10 (70%), Positives = 8/10 (80%), Gaps = 0/10 (0%)
Frame = +1

Query  55   LLLSWLGGKE  64
            LLLS  GGK+
Sbjct  448  LLLSMWGGKK  477
```

```
>ndhH
Length = 1123

Score = 18.1 bits (35),  Expect = 4.9, Method: Compositional matrix adjust.
Identities = 8/20 (40%), Positives = 11/20 (55%), Gaps = 0/20 (0%)
Frame = +3

Query  46   TGILLAMHYVGHVDYAFASV  65
             G L   +Y G   Y+F+SV
Sbjct  288  KGQLYQSNYAGIESYSFSSV  347

Score = 16.2 bits (30),  Expect = 4.1, Method: Composition-based stats.
Identities = 8/19 (42%), Positives = 11/19 (58%), Gaps = 0/19 (0%)
Frame = -1

Query  41   ILTEKLFGAFLADVLLLSW  59
            I  EKL+ +  A +L  SW
Sbjct  349  ITDEKLYDSIPA*LL*YSW  293
```

Pinaceae

Picea abies

```
>ndhD
Length = 1503

Score = 20.0 bits (40), Expect = 1.8, Method: Compositional matrix adjust.
Identities = 39/155 (25%), Positives = 63/155 (41%), Gaps = 35/155 (23%)
Frame = +1

Query  301  IATMWGGSIRY----KTPMLSAAGSIFPSTIGGLTGIVLA---NPGLDIALHDTHYVVAH  353
            + +MWGG  R     K  + +A  SIF   + G+ GI L     P L++ L      +A+
Sbjct  451  LLSMWGGKKRLYSATKFILYTAGSSIF--LLIGVLGISLYGSNEPTLNLEL------LAN  606

Query  354  SHYVLSMGAVFASSAGSHFRVGK--IPGRTY-PETLGQIHLRITLFGVNSTFFPMHFLGL  410
              Y +++  +F       F V    IP  T+ P+T G+ H    +              L
Sbjct  607  KSYPVTLEILFYIGFLIAFAVKSPIIPLHTWLPDTHGEAHYSTCML-------------L  747

Query  411  SGMPRRIPDYPDAYAGWNAL----SSFGPYVSVVG  441
            +G+  ++  Y         L    S F P++ VVG
Sbjct  748  AGILLKMGAYGLVRINMELLPHAHSMFSPWLLVVG  852
```

Pinus taeda

```
>ndhD
Length = 1503

Score = 18.9 bits (37), Expect = 4.6, Method: Compositional matrix adjust.
Identities = 16/49 (33%), Positives = 24/49 (49%), Gaps = 5/49 (10%)
Frame = +3

Query  416  RITLFGVNSTFFPMHFLGLSGMPRRIPDYPDAYAGWNALSSFGPYVSVV  464
            RI  F  +STFF  H +G+  +  R+  +   + GW   S FG  +  V
Sbjct  378  RIIFFSGSSTFF--HHVGIR-INSRLSTF--IHVGWKETSVFGYKIYFV  509
```

```
Score = 18.1 bits (35), Expect = 6.6, Method: Compositional matrix adjust.
Identities = 44/179 (25%), Positives = 70/179 (39%), Gaps = 36/179 (20%)
Frame = +1

Query  325  IATMWGGSIRY----KTPMLLAAGSIFPSTIGGLTGIVLA---NPGLDIALHDTHYVVAH  377
            + +MWGG  R     K  +  A  SIF   + G+ GI L     P L++ L      +A+
Sbjct  451  LLSMWGGKKRLYSATKFILYTAGSSIF--LLIGVLGISLYGSNEPTLNLEL------LAN  606

Query  378  SHYVLSMGAVSASSAGFHFRVGK--IPGRTY-PETLGQIHLRITLFGVNSTFFPMHFLGL  434
              Y +++  +        F V    IP  T+ P+T G+ H    +              L
Sbjct  607  KSYPVTLEILFYIGFLIAFAVKSPIIPLHTWLPDTHGEAHYSTCML-------------L  747

Query  435  SGMPRRIPDYPDAYAGWNAL----SSFGPYVSVVGIFRFFVVVTITLGSGN-NKRCAPS  488
            +G+  ++  Y         L    S F P++ VVG  +     + + G  N  KR A S
Sbjct  748  AGILLKMGAYGLVRINMELLPHAHSMFSPWLLVVGTIQIIYAASTSPGQRNLKKRIAYS  924

Score = 18.1 bits (35), Expect = 7.0, Method: Compositional matrix adjust.
Identities = 6/14 (43%), Positives = 11/14 (79%), Gaps = 0/14 (0%)
Frame = +3

Query  261  STFSGKPVFGYPGM  274
            S++SG P+F +P +
Sbjct  315  SSYSGFPIFSFPDV  356
```

Pinus banksiana

```
>ndhD
Length = 1503

Score = 18.1 bits (35), Expect = 5.2, Method: Compositional matrix adjust.
Identities = 6/14 (43%), Positives = 11/14 (79%), Gaps = 0/14 (0%)
Frame = +3

Query  195  STFSGKPVFGYPGM  208
            S++SG P+F +P +
Sbjct  315  SSYSGFPIFSFPDV  356
```

Abies balsamea

```
>ndhH
Length = 1123

Score = 17.3 bits (33), Expect = 3.6, Method: Compositional matrix adjust.
Identities = 7/22 (32%), Positives = 9/22 (41%), Gaps = 0/22 (0%)
Frame = -1

Query  120  PSTIGGLTGIVLANPGLDIAPH  141
            P        +V  NP L + PH
Sbjct  694  PMVFRMRVALVRINPNLLLLPH  629
```

Pinus koraiensis

```
>ndhD
Length = 1503

Score = 18.1 bits (35), Expect = 5.3, Method: Compositional matrix adjust.
Identities = 6/14 (43%), Positives = 11/14 (79%), Gaps = 0/14 (0%)
Frame = +3

Query  217  STFSGKPVFGYPGM  230
            S++SG P+F +P +
Sbjct  315  SSYSGFPIFSFPDV  356
```

Pinus thunbergii

```
>ndhD
Length = 1503

Score = 18.1 bits (35), Expect = 5.2, Method: Compositional matrix adjust.
Identities = 6/14 (43%), Positives = 11/14 (79%), Gaps = 0/14 (0%)
Frame = +3

Query  195  STFSGKPVFGYPGM  208
            S++SG P+F +P +
Sbjct  315  SSYSGFPIFSFPDV  356
```

SUPPLEMENTARY FIGURE 4 | Sequence alignment between the mitochondrial genome of Chlorophyceae and Pinaceae and the *ndhF-D-E-G-I-A-H* region of *Arabidopsis thaliana*.

SUPPLEMENTARY TABLE 1 | List of species and GenBank accession numbers.

Species	Accession Numbers
**Archaea**	
Methanosarcina barkeri	NZ_CP009530
Methanocorpusculum labreanum	NC_008942
Methanospirillum hungatei	NC_007796
Methanosphaerula palustris	NC_011832
Methanoculleus marisnigri	NC_003901
Methanosarcina mazei	NC_003901
**Cyanobacteria**	
Thermosynechococcus elongatus BP-1	NC_004113
Synechocystis sp. PCC 6803	NC_000911
Nostoc punctiforme PCC 73102	NC_010628
Nostoc sp. PCC 7120	NC_003272
Trichormus variabilis ATCC 29413	NC_007413
Synechococcus elongatus PCC7942	NC_007604
**Chlorophyceae**	
Chlamydomonas reinhardtii	NC_005353
Volvox carteri	GCF_000143455
Dunaliella salina	NC_016732
Tetradesmus obliquus	NC_00810
Gonium pectorale	NC_020438
Chromochloris zofingiensis	NC_024758
**Bryophyta**	
Tetraphis pellucida	NC_024291
Sanionia uncinata	NC_025668
Anthoceros formosae	NC_004543

Sphagnum lescurii	KU725457
Marchantia polymorpha	NC_042505
Physcomitrella patens	NC_005087
**Pinaceae**	
Picea abies	NC_021456
Pinus taeda	NC_021440
Pinus banksiana	FJ899571
Abies balsamea	NC_042778
Pinus koraiensis	NC_004677
Pinus thunbergii	NC_001631
**Monocots**	
Zea mays	NC_001666
Sorghum bicolor	NC_008602
Oryza sativa	NC_001320
Asparagus officinalis	NC_034777
Zostera marina	NC_036014
Phoenix dactylifera	NC_013991
Hordeum vulgare	NC_056985
**Dicots**	
Solanum lycopersicum	NC_007898
Arabidopsis thaliana	NC_000932
Daucus carota	NC_008325
Vigna radiata	NC_013843
Capsella rubella	NC_027693
Ricinus communis	NC_016736
Nicotiana attenuata	NC_035952
